# Evidence of neurofibromatosis type 1 in a multi-morbid Inca child mummy: A paleoradiological investigation using computed tomography

**DOI:** 10.1371/journal.pone.0175000

**Published:** 2017-04-12

**Authors:** Stephanie Panzer, Holger Wittig, Stephanie Zesch, Wilfried Rosendahl, Sandra Blache, Magdalena Müller-Gerbl, Gerhard Hotz

**Affiliations:** 1 Department of Radiology, Trauma Center Murnau, Murnau, Germany; 2 Institute of Biomechanics, Trauma Center Murnau and Paracelsus Medical University Salzburg, Murnau, Germany; 3 Institute of Legal Medicine, University of Basel, Basel, Switzerland; 4 German Mummy Project, Reiss-Engelhorn-Museen Mannheim, Mannheim, Germany; 5 Anatomy, University of Basel, Basel, Switzerland; 6 Anthropology, Natural History Museum of Basel, Basel, Switzerland; Seoul National University College of Medicine, REPUBLIC OF KOREA

## Abstract

**Objective:**

In this study, an Inca bundle was examined using computed tomography (CT). The primary aim was to determine the preservation status of bony and soft tissues, the sex, the age at the time of death, possible indicators for disease or even the cause of death, as well as the kind of mummification. A secondary aim was to obtain a brief overview of the wrapping in order to gain additional information on the cultural background.

**Materials and methods:**

The bundle belongs to the Museum of Cultures in Basel, Switzerland, and was bought in Munich, Germany, in 1921. Radiocarbon dating of the superficial textile yielded a calibrated age between 1480 and 1650 AD. The mummy was investigated using multi-slice CT with slice thickness of 0.75 mm and 110 kilovolt. For standardized assessment of soft tissue preservation, a recently developed checklist was applied.

**Results:**

CT revealed the mummy of a seven to nine year old boy with superior preservation of bony and soft tissues allowing detailed assessment. Indicators of neurofibromatosis type 1 (paravertebral and cutaneous neurofibromas, a breast neurofibroma, sphenoid wing dysplasia), Chagas disease (dilatation of the esophagus, stomach, rectum, and large amounts of feces), and lung infection (pleural adherence, calcifications), probably due to tuberculosis, were found. Furthermore, signs of peri-mortem violence (transection of the chest and a defect in the abdominal wall) were detected. CT images revealed a carefully performed wrapping.

**Conclusion:**

CT examination of the Inca bundle proved to be an important non-destructive examination method. Standardized assessment, especially of the soft tissue structures, allowed for diagnoses of several diseases, indicating a multi-morbid child at the time of death. The careful wrapping pointed to a ceremonial burial. Within the cultural background, the signs of fatal violence were discussed as a possible result of war, murder, accident, or human sacrifice.

## Introduction

Bioarchaeology (the scientific study of human remains from archaeological sites) provides insight into the biological status, including diseases, of past people [1]. Paleoradiology is the study of bioarchaeological materials using modern imaging methods, such as X-ray radiography, computed tomography (CT), magnetic resonance imaging, and micro-CT [2]. The first CT examination of an Egyptian mummy was reported in 1979 [3] and since then CT developed into the “gold-standard” for human mummy studies due to its non-destructive nature, high spatial resolution, image contrast, and the possibility of different post-processing modalities [4–12].

Soft tissues are the substances that make a skeleton into a mummy and they allow for a diagnosis beyond osteology [13]. In contrast to bones, soft tissues undergo a distinct postmortem change during the mummification process mainly due to tissue dehydration [13]. Soft tissues are known to have a different appearance on CT images in comparison to living patients regarding density, size and shape, and sometimes location within the body. Therefore, correct image interpretation is often complicated [14–19].

In clinical radiology, structured reporting is increasingly being established. That means a diagnosis is made following templates or checklists [20–23]. One such approach in paleoradiology is the recently developed “Checklist and Scoring System for the Assessment of Soft Tissue Preservation in CT Examinations of Human Mummies” [14]. The checklist serves as a tool to guide the radiologist through the main soft tissues structures throughout the body and enables a standardized checkpoint acquisition. Thereby, assessment of the preservation status of the soft tissues is considered to be the starting point for detection of possible pathological findings of these tissues [14].

As medical history and physical examination are not available for paleopathological studies, knowledge of the cultural environment, such as diet, typical diseases, general life circumstances or mummification and ritual practices, may provide additional information [13, 24]. Therefore, pathological or conspicuous radiological findings should be interpreted within the cultural history [4, 7, 12, 25–27].

In this study, CT was applied to an Inca mummy bundle from the Museum of Cultures in Basel, Switzerland. CT was believed to be the best non-destructive imaging method to gain information about the body, which was completely wrapped in thick layers of textile and therefore, was not accessible without unwanted destruction of the wrapping.

The aim of this study was to determine the preservation status of bony and soft tissues, sex of the mummified individual, age at the time of death, possible indicators for disease or even the cause of death, as well as the kind of mummification. A secondary aim was to obtain a brief overview of the wrapping in order to gain any additional information on the cultural background of the bundle.

## Materials and methods

### Material

The bundle belongs to the Museum of Cultures in Basel, Switzerland, where it is also housed. In 1921, the bundle was bought by Dr. Franz Xaver Weizinger (1883–1945) in Munich. He was a well-known German art historian and auction trader who had good contacts in South America. The provenance of the bundle before its disposal in Munich is still unknown.

**Fig 1:**
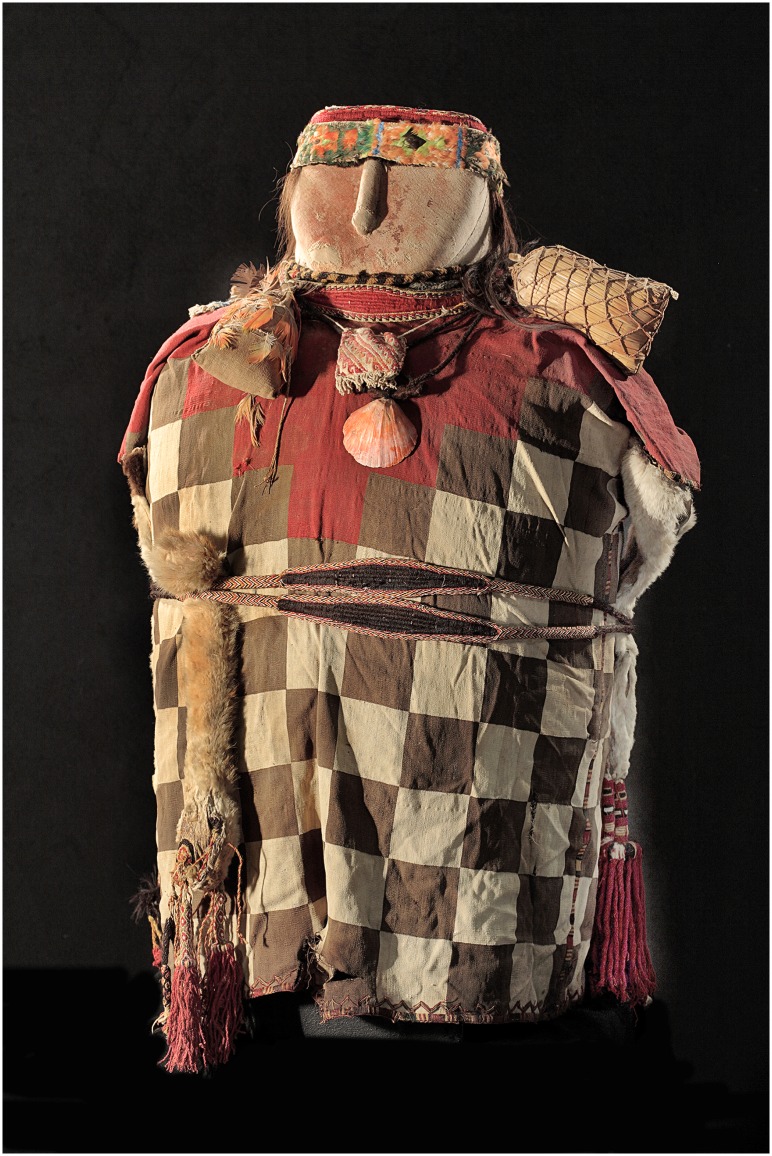
Photograph of the mummy. Frontal view of the bundle showing a false head decorated with a nose, a wig made of human hair with two thin braids, and a woven cap with ornaments. The body was dressed in a typical Inca tunic with a plaid pattern in different colors. The dress contained a collar and small bags fixed around the neck. A necklace with a reddish spondylus shell also hung around the neck. Bags, made from the fur of llama legs, are strung around the body using two thick cords. Radiocarbon dating (Klaus-Tschira Archaeometry Centre, Mannheim) yielded an AMS-^14^C age of 311 ± 35 years BP for a sample of the mummy’s tunic textiles (laboratory number MAMS 27250) and a calibrated age between 1480 and 1650 years cal AD (95.4% confidence interval).

The cylindrical bundle had a maximum cross section of about 50 cm and a height of approximately 71 cm. On top of the body was a slightly conical false head. The body was dressed in a typical Inca tunic. Four small bags hung around the neck and a necklace with a spondylus shell was present. A slingshot was bound around the body and another smaller one around the neck. Three bags, made from of the fur of llama legs, strung around the waist completed the setting (Fig 1).

### Methods

Permission for the CT examination of the mummy (collection number IVc 2813) was granted by the Museum of Cultures in Basel, Switzerland.

The mummy was investigated using a multi-slice CT (SIEMENS Emotion 16; slice thickness 0.75 mm, voltage 110 kilovolt) at the Institute of Legal Medicine, University of Basel, Switzerland. Additional reconstructions were performed at the CT workstations of the involved institutions, such as multiplanar reconstructions, maximum intensity projections, and three dimensional reconstructions.

For the assessment of soft tissue preservation, a recently developed checklist [14] was applied.

Age at the time of death of the mummy was determined based on tooth development and eruption [28], fusion of skull sutures [29], maximum length of second metacarpals [30], and length of long hollow bones [31].

## Results

### Preservation status

**Fig 2:**
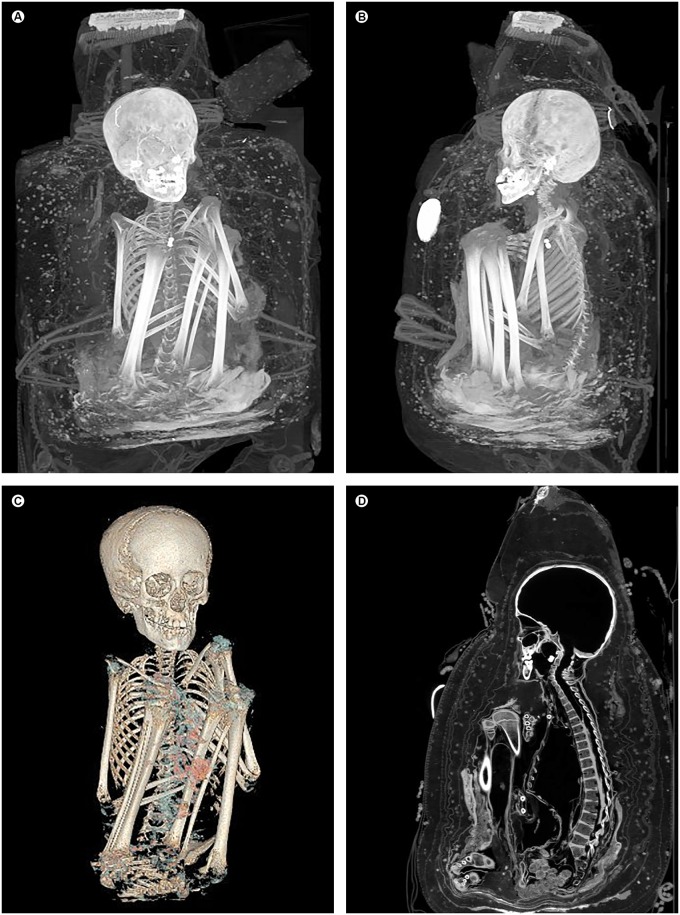
Overview of the skeleton inside the bundle. (A, B) Maximum intensity projections, (C) three-dimensional reconstruction, and (D) sagittal multiplanar reconstruction showing the skeleton in a squatting position with tightened legs, flexed knees and partially crossed interiorly rotated and adducted feet. Both arms were located between the body and the legs with flexion of the elbows. Note the kyphoscoliosis of the lower thoracic and the lumbar spine.

The skeleton was completely preserved. The individual was in a squatting position with tightened legs and flexed knees. Both arms were located between the body and the legs. Some minor, presumably post-mortem, dislocations of skeletal elements were present. Otherwise, articulation and alignment were intact (Fig 2). Three maxillary teeth and the fragment of another tooth were dislocated to the throat and the esophagus. Cartilage was preserved throughout the skeleton.

Based on the “CT Checklist and Scoring System”, soft tissue preservation revealed a total score of 137.5 (out of 200), consisting of 89 (out of 100) for the main category “Soft Tissues of Head and Musculoskeletal System” and 48.5 (out of 100) for the main category “Organs and Organ Systems”. Preserved soft tissues of the head included the nose, auricles, ossicles, eye bulb and/or lens, eye muscles, optic nerve, falx, and tentorium. Soft tissues of the musculoskeletal system included: tendons and/or musculature of different sites of the neck and trunk as well as the upper and lower extremities; peri- and intraarticular soft tissues such as the rotator cuff and the capsule and/or labrum of the shoulder, the capsule and/or labrum of the hips, and the posterior cruciate ligament and the medial and lateral meniscus of the knees. Additionally, intervertebral discs of the thoracic and lumbar spine were preserved. From the central nervous system, the brain was preserved in form of undefined remnants in the middle cranial fossa. The spinal cord was detectable in the cervical and thoracic spine. Peripheral nerves were detectable in the entire spine. From the cardiorespiratory system, slight remnants of the hypopharynx and larynx were preserved. The trachea and both lungs were preserved. The upper portion of the heart was discernible consisting of parts of the pericardium, all four chambers, the intraventricular septum, and the aortic valve. Parts of the diaphragm were visible on both sides. From the gastrointestinal system, the tongue, esophagus, stomach, intestine, rectum and/or anus, and the liver were preserved. From the genitourinary, system both kidneys and the penis were discernible. Arteries could be found in the neck and mediastinum.

### Age estimation

**Fig 3:**
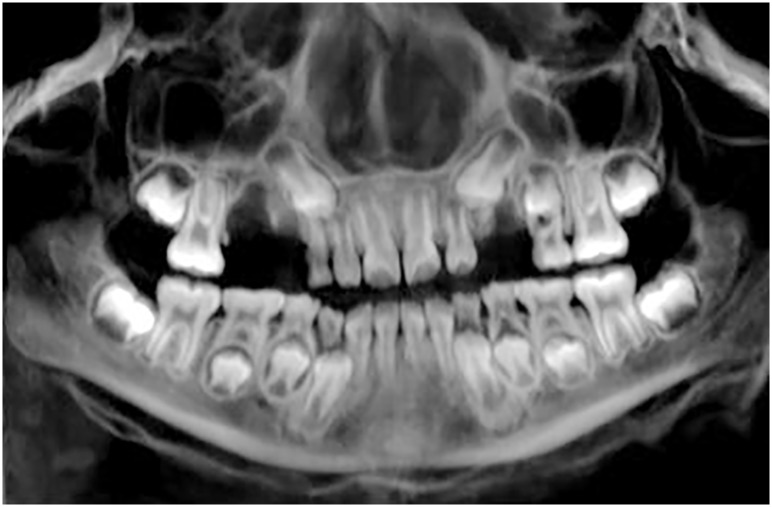
Dentition. Curved multiplanar reconstruction of the upper and lower jaw (the right side in the reconstruction corresponds to the right side in the mummy) gives an overview of the dentition. Several deciduous teeth were still in the jaw including the upper left canine and upper right second molar, as well as the lower canines and lower molars. The permanent incisors and permanent first molars had already erupted, while the permanent second molars had not yet erupted. The crowns of all permanent canines, the upper right second premolar, and all lower premolars were observed inside the jaw. The germs of the third molars were not yet mineralized. Seven maxillary teeth were present including deciduous teeth (first molars and left second molar) and permanent teeth (tooth crowns of first premolars and left second premolar); three of them and a tooth fragment were dislocated.

The determination of age at death was conducted by assessing tooth development and tooth eruption, as well as various skeletal markers. Several deciduous teeth were still in the jaw, however, the permanent incisors and permanent first molars had already erupted indicating an age between seven and eight years (Fig 3) [28]. Fused sutura intra-occipitalis anterior and posterior suggested an age older than seven [29]. The maximum length of second metacarpals [30] indicated an age from six to seven, while the length of long bones [31] suggested an age from seven to nine years [31] which was finally deemed to be a reliable age if taking into account all mentioned developmental markers.

### Pathological findings indicating neurofibromatosis type 1

**Fig 4:**
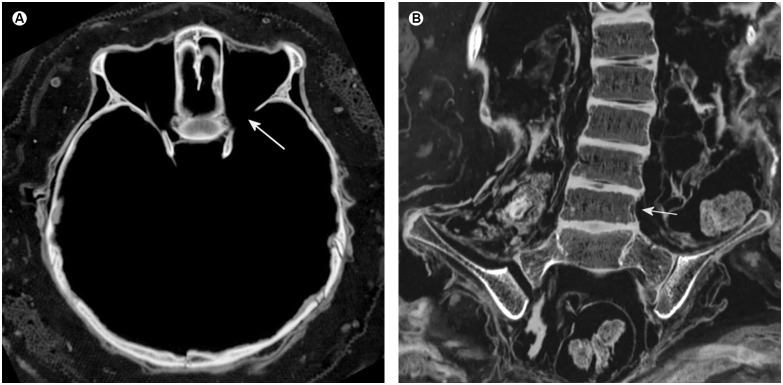
Osseous findings indicating neurofibromatosis type 1. (A) Axial multiplanar reconstruction of the basal skull demonstrating a slight sphenoid wing dysplasia on the left side (arrow). (B) Coronal multiplanar reconstruction of the lower thoracic spine and the lumbar spine showing scoliosis and discrete wedging of the body of the fifth lumbar vertebra in the frontal plane with reduced height on the left side (arrow). Note the preservation of the intervertebral discs.

Skeletal abnormalities indicative of neurofibromatosis type 1 (NF 1) consisted of a slight sphenoid wing dysplasia on the left side (Fig 4A), a moderate kypho-scoliosis of the lower thoracic spine and the lumbar spine (Figs 1 and 4B), slightly hypoplastic dorsal elements of the fourth and fifth lumbar vertebra, enlargement of a lumbar neuroforamen (see below) and discrete wedging of the body of the fifth lumbar vertebra (Fig 4B).

**Fig 5:**
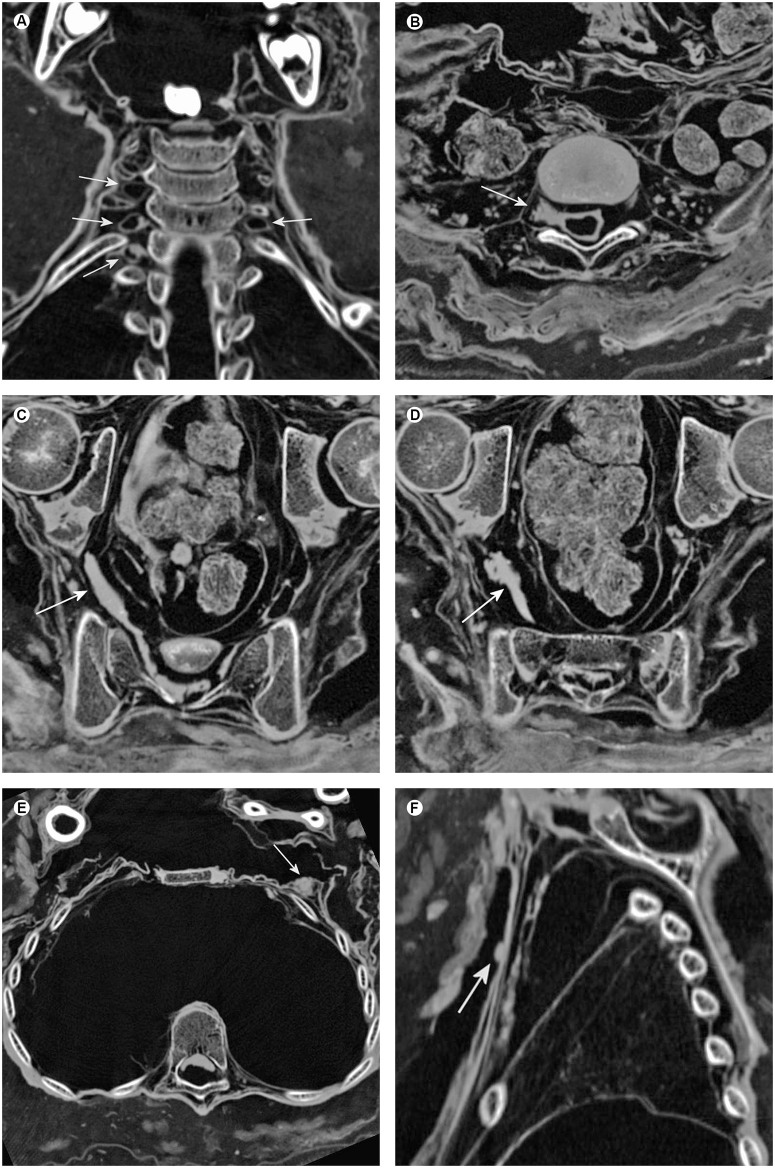
Soft tissue findings indicating neurofibromatosis type 1. (A) Coronal multiplanar reconstruction of the neck illustrating probable cystic neurofibromas at the cervico-thoracic junction (arrows). (B) Axial multiplanar reconstruction of the lumbo-sacral junction displaying an assumed neurofibroma in the course of the fifth lumbar spinal nerve root (arrow) and a slight enlargement of the neuroforamen. Note multiple small nodules in the paravertebral space probably indicating neurofibromas. (C, D) Axial multiplanar reconstructions of the pelvis showing a supposed plexiform neurofibroma within the right sacral plexus (arrows). (E) Axial multiplanar reconstruction of the chest illustrating a supposed breast neurofibroma on the left side (arrow). (F) Sagittal multiplanar reconstruction of the chest revealing an assumed cutaneous neurofibroma (arrow).

Several soft tissue findings indicated the presence of NF 1. Paravertebrally at the cervico-thoracic junction, four small round masses (maximum diameter of 6 mm) with hypodense centers were detectable coming from the spinal nerve roots in the extraforaminal course (Fig 5A). A lobulated mass (maximum length of 10 mm) was recognizable in the intra- and extraforaminal course of the right fifth lumbar spinal nerve root. The right neuroforamen was slightly enlarged in this segment (Fig 5B). Within the right sacral plexus a plexiform mass (maximum extent of 30 x 30 mm) deriving from the first and second sacral spinal nerve roots was present (Fig 5C and 5D). Multiple small nodules were found in the paravertebral spaces of the lumbar and sacral spine as well as at the neck (maximum diameter of 6 mm). A lobulated mass was detected in the region of the left breast (maximum diameter of 16 mm) (Fig 5E). The surface of the scalp appeared to be knobby and on or within the cutis of the body some small nodules (size of 3–4 mm) were identified (Fig 5F).

### Pathological findings indicating Chagas disease

**Fig 6:**
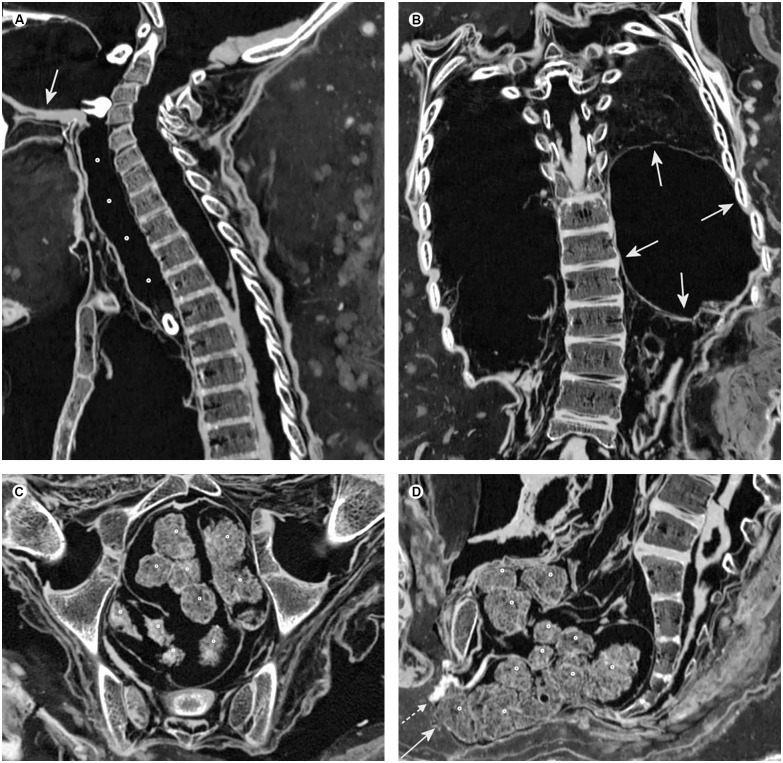
Soft tissue findings indicating Chagas disease. (A) Sagittal multiplanar reconstruction of the neck demonstrating dilation of the esophagus in its entire course (circles). Note the dislocation of three teeth inside the oral cavity and the esophagus as well as preservation of the tongue (arrow). (B) Coronal multiplanar reconstruction of the chest and abdomen revealing distinct enlargement of the stomach (arrows) with superior displacement of the left lung. (C) Axial multiplanar reconstruction of the pelvis showing the dilation of the rectum filled with feces (circles). (D) Sagittal multiplanar reconstruction of the pelvis illustrating dilation of the pelvis and distinct accumulation of feces (circles) bulging out at the anus (arrow). Note the preservation of the penis directly overlying the rectum (dotted arrow).

CT images revealed dilation of the esophagus in its entire course with a maximum diameter of 2 cm (Fig 6A). The transition from the esophagus into the stomach was largely preserved. The stomach seemed to be present with an enlargement up to 10 cm in the cranio-caudal direction and 9 cm in the antero-posterior direction filling up large parts of the left thoracic and abdominal cavities (Fig 6B). Inside the abdomen and pelvis, distinct remnants of feces were present in the form of round to oval partially coherent coprolites with a maximum diameter of 2.6 cm. They were found especially inside the cecum with dilatation of the lumen up to 3.6 cm and in the descendent and sigmoid colon with dilatation up to 4.6 cm. The rectum was filled up with feces that extended the lumen up to 4.3. cm in diameter and bulged out at the anus (Fig 6C and 6D).

### Pathological findings indicating lung infection

**Fig 7:**
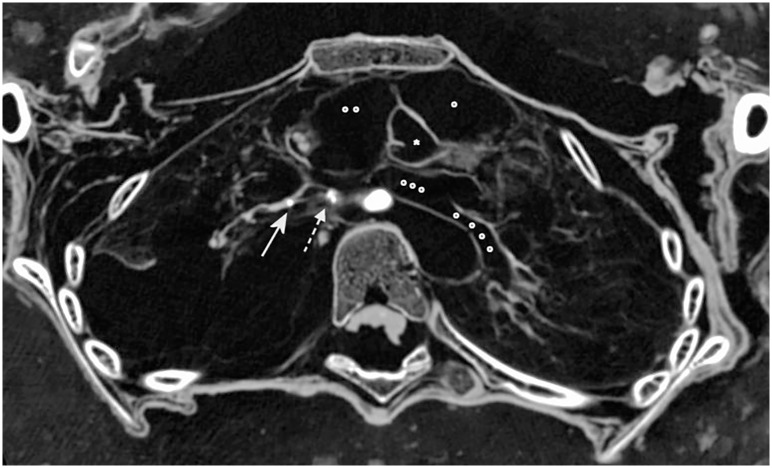
Pathological findings of the lungs. Axial multiplanar reconstruction of the chest illustrating one of the three intraparenchymatous calcifications of the right lung (arrow) and calcification in the region of the right hilum (dotted arrow). Both upper lobes and parts of the left lower lobe revealed broad contact to the chest wall. Note the distinct lung preservation with discernible parenchyma, vessels and airways. The heart was displaced superiorly and medially with retrosternal location. Note the preservation of the aortic valve (asterisk), parts of the right ventricle (circle), the right atrium (two circles) and the left atrium (three circles) with an originating pulmonal vein (four circles).

Both upper lobes of the lung showed only slight collapse and had broad contact to the pleura respective to the chest wall. The right middle, and lower lobe, as well as the left lower lobe were partially collapsed. On the right side, three small calcifications were detected within the parenchyma of the lung and another calcification with maximum diameter of 3 mm was found within the region of the right hilum (Fig 7).

### Pathological findings possibly indicating peri-mortem violence

**Fig 8:**
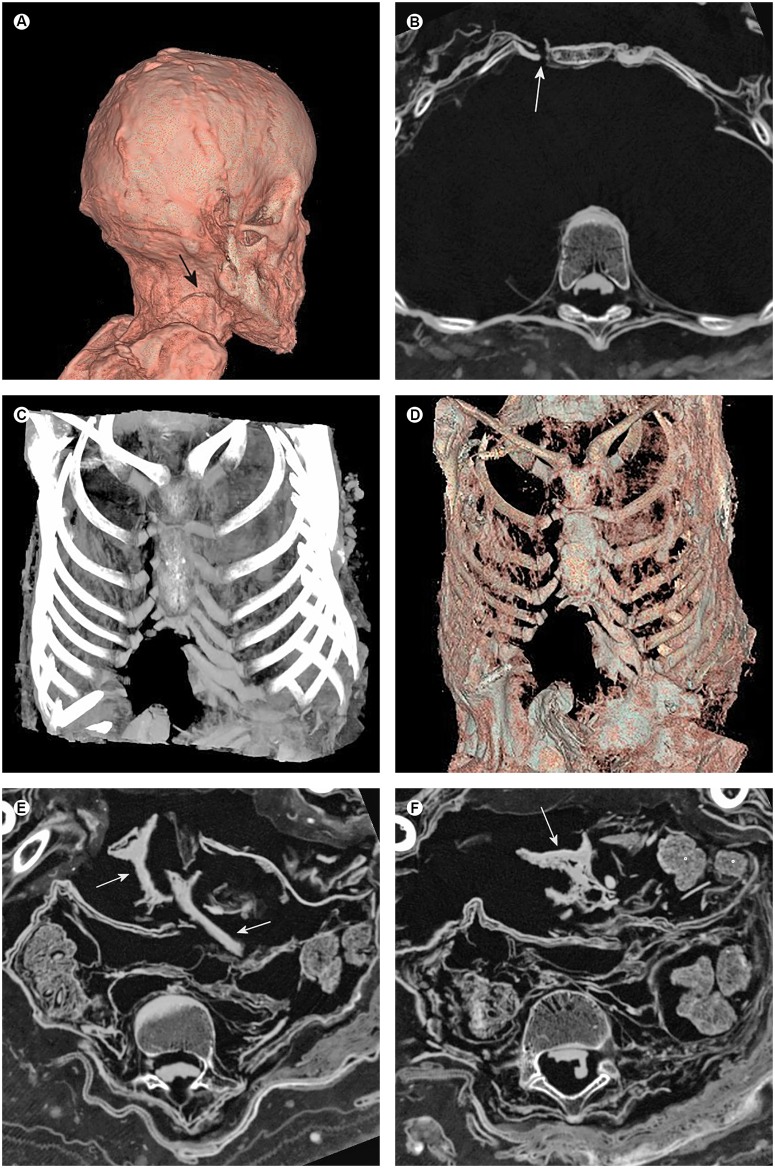
Signs of possible peri-mortem violence. (A) Three-dimensional reconstruction of the head and neck demonstrating a slightly curved discontinuity of the cutis at the right side of the neck (arrow). Note the knobby surface of the scalp possibly indicating cutaneous neurofibromas. (B) Axial multiplanar reconstruction of the chest showing a slightly dehiscent sharp transection of the third rib within the cartilage near the sternum on the right side (arrow). (C) Maximum intensity projection of the chest illustrating the transection of the second to sixth rib on the right side, as well as the loss of the common cartilaginous insertion of the seventh and eight ribs. On the left side, a cut through the common cartilaginous insertion of the seventh and eight ribs was visible. (D) Three-dimensional reconstruction of the chest and abdomen demonstrating a large defect of the abdominal wall adjacent to the thoracal transection. (E, F) Axial multiplanar reconstructions of the abdomen illustrating the defect and displacement of the abdominal wall, as well as dislocation of the fragmented liver (arrows) and several coprolites (circles) into the extra-abdominal space.

On the right side of the neck, an approximately 5 cm long curved discontinuity of the cutis without dehiscence was recognizable ranging from underneath the chin to the dorsal part of the neck (Fig 8A).

On the anterior chest, on the right side next to the sternum, a transection of the cutis and the underlying cartilage of the second to sixth rib were recognizable with different extents of dehiscence (Fig 8B and 8C). The transection led directly into a large defect of the anterior abdominal wall (maximum extent of approximately 5 x 8 cm) ranging to the height of the ilium (Fig 8D). In the anteroposterior direction, the abdominal wall was displaced up to 3.5 cm. Pieces of feces, as well as the fragmented liver, erupted through the defect in the abdominal wall (Fig 8E and 8F).

Fractures without dislocation and without callus formation were found in the left distal humerus and the left distal tibia.

### Wrapping and mummification

**Fig 9:**
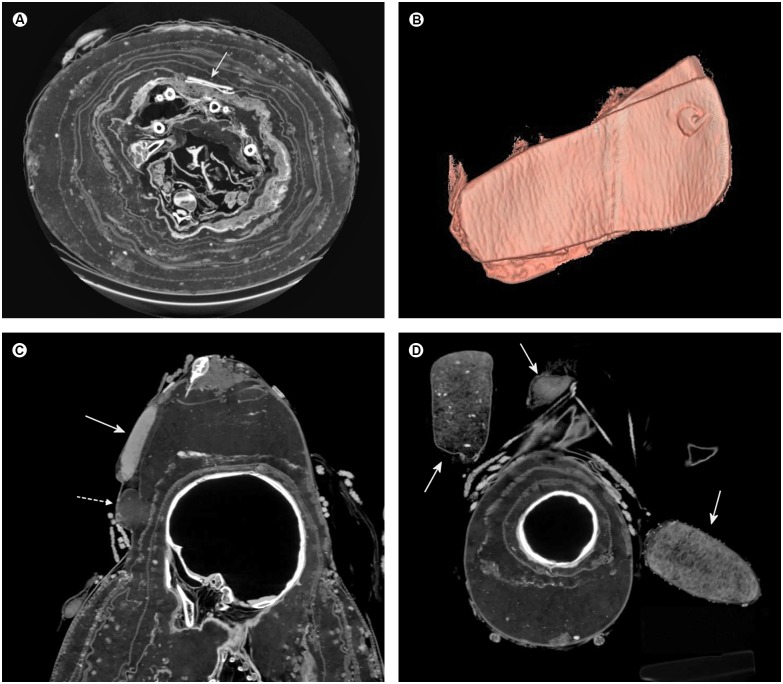
Wrapping. (A) Axial multiplanar reconstruction of the bundle illustrating different layers of textiles with interworked cord structures around the mummy. In the front of the lower legs, two thin hyperdense objects were found (arrow) overlying the inner hyperdense textile layer. (B) Three-dimensional reconstruction of these objects displaying the form of shoe soles and a kind of loop in the region of the big toe. (C) Sagittal multiplanar reconstruction showing the composition of the false head with a hyperdense textile roll forming the nose (arrow) and another textile roll forming the chin (dotted arrow). (D) Paraaxial multiplanar reconstruction illustrating the different contents of the bags around the neck (arrows).

The mummy was seated on layers of hyperdense thick materials, which partially surrounded the feet and the lower part of the pelvis and was then fixed by cords. Several alternating layers of textiles were wrapped around the body with tightly interworked cord structures. Additional packing materials seemed to have been used resulting in multiple small hyperdensities (Fig 2A, 2B and 2C). The wrapping revealed a maximum thickness of 16 cm in the region of the body. Two thin hyperdense structures with the form of shoe soles were found in front of the lower legs overlying the deep hyperdense textiles (Fig 9A and 9B). They revealed a kind of loop in the region of the big toe (Fig 9B). The false head was reconstructed with additional textiles formed as rolls (Fig 9C). The small bags around the neck each included different materials, based on the structure and density (Fig 9D). No signs of artificial mummification were found within the body itself.

## Discussion

CT examination of this child mummy allowed for age estimation at the time of death, detailed assessment of the preservation status, especially of the soft tissues, and diagnoses of diseases. Furthermore, distinct signs of violence led to speculations on the circumstances of the child’s death within its cultural environment.

### Neurofibromatosis type 1

Pathologies found in the child mummy fulfill the criteria for the clinical diagnosis of neurofibromatosis type 1 (NF 1), also known as von Recklinghausen disease. Table 1 shows a variety of abnormalities resulting from NF 1 in the clinical presentation [32] and summarizes the presence and absence of these findings on CT images of the mummy. To make the diagnosis, two or more criteria must be met. In this case, the requirement was fulfilled by the paravertebral neurofibromas at the cevico-thoracic junction and the lumbar spine, the neurofibroma of the left breast, as well as the sacral plexiform neurofibroma and the sphenoid wing dysplasia. Thus, the diagnosis could be made, even without the possibility to assess externally visible pathologies such as: café au lait spots; axillary or inguinal freckling: or iris hamartomas, and without the possibility to ask for primary relatives with NF 1 [32–35].

**Table 1:** Variety of abnormalities in NF1.

Variety of abnormalities in NF1:	Abnormalities detected on CT images of the child mummy
**Osseous pathologies detectable on CT images**	
Distinctive osseous lesions:	Detected
•Kyphoscoliosis	Detected
•Posterior vertebral scalopping	Not detected
•Hypoplastiv posterior elements	Detected
•Vertebral wedging	Detected
•Enlarged neural foramina	Detected
•Ribbon rib deformity, rib notching and dysplasia	Not detected
•Tibial pseudarthrosis or less commonly ulnar pseudarthrosis	Not detected
•Bony dysplasia: especially affecting tibia	Not detected
•Severe bowing, gracile bones	Not detected
•Multiple non-ossifying fibromas	Not detected
•Limb hemihypertrophy	Not detected
•Lambdoid suture defect	Not detected
Sphenoid wing dysplasia	Detected
**Soft tissue pathologies detectable on CT images**	
Two or more neurofibromas or one plexiform neurofibroma	Detected
Neurofibroma of the breast	Detected
Optic nerve glioma (only larger ones are detectable on CT)	Not detected
Dural ectasia	Not detected
Cutaneous and subcutaneous neurofibromas	Detected
Mediastinal mass	Not detected
Lung parenchymal disease	Not detected
Vascular pathologies	Not detected
Neoplasms	Not detected
**Pathologies not detectable on CT images**	
≥6 Cafe au lait spots evident during one year	
Two or more iris hamartomas (Lisch nodules)	
Axillary or inguinal freckling	
Primary relative with NF1	

To make the clinical diagnosis, two or more of the highlighted abnormalities are required.

NF 1 is a multisystemic neurocutaneous disorder and the most common phakomatosis [32]. NF 1 affects 1:2500–3000 individuals [36]. In half of these cases, the disease is inherited as an autosomal dominant condition and in the other half of cases it is due to a new mutation [32, 37]. The NF 1 gene locus is on chromosome 17 [32, 37]. NF 1 has an early age of onset with approximately 50% of patients meeting the diagnostic criteria by the age of one year and approximately 97% of patients meeting the criteria by the age of eight years [32, 38]. Although the prognosis is variable, patients with NF 1 have a life expectancy approximately half that of non affected individuals, usually succumbing to tumors or cardiovascular complications [32, 39].

To the best of our knowledge, this is the first definite diagnosis of NF 1 in paleopathological and paleoradiological literature based mainly on soft tissue findings in CT images. In paleopathological literature, few cases consider the diagnosis of NF 1 in skeletal remains [40–42]. Probable historical cases of NF 1 are discussed in the Ebers Papyrus Case #873 from Egypt dated at 1536 BC [43], a statue from Hellenistic times [44], the kings of Parthia (247 BC– 224 AD) as depicted on coins [45], and the painting “La Camera degli Sposi” from Andrea Mantegna (1431–1506) in Italy [46]. Frederick von Recklinghausen is credited with the discovery of NF 1 and coined the name of the disorder in 1882 [47].

### Chagas disease

The dilation of the esophagus, stomach and colon (the so called “megaviscera”) with massive amounts of feces indicated the diagnosis of chronic Chagas disease in this case. Chagas disease is a chronic, systemic, parasitic infection caused by the protozoan Trypanosoma cruzi and was discovered by the Brazilian physician, bacteriologist and epidemiologist Carlos Chagas in 1909 [48, 49]. Currently, the disease affects about 8 million people in Latin America, of whom 30–40% either have or will develop cardiomyopathy, digestive megasyndroms, or both [48]. The chronic form usually develops 10–30 years after the initial infection and is divided into a cardiac, a cardiodigestive and a digestive form [48]. The mummy showed signs of the digestive form. As the heart was only partially preserved, possible signs of the heart being affected were not sufficiently assessable. Therefore, a cardiodigestive form could neither be confirmed nor excluded. Megaesophagus is a common finding in chronic Chagas disease and dysphagia may lead to malnutrition and severe weight loss. Megastomach is rather rare and can lead to alteration in motility and secretion. Megacolon is another common finding and often produces prolonged obstipation, abdominal distension, and occasionally large bowel obstruction [48, 49]. Due to the young age of the mummy referring to an already chronic form of the disease, a congenital infection could be assumed [49].

In the paleopathological literature, Chagas disease is well known and documented by macroscopy, histology, immunhistochemistry, Trypanosoma cruzi DNA extraction, and radiology [25, 50–55]. The animal-infected cycle of Chagas disease was probably already well established 9,000 years ago. At this time, the earliest humans (members of the Chinchorro culture) first peopled costal and low valley sites in northern Chile and south Peru and inadvertently joined the many other species acting as hosts for this parasite [50].

The differentiation between hollow organ dilation as a result of post-mortem bacterial growth with gas formation and intra-vital development of organ dilation has already been discussed. Distension of the rectum with feces and a colon packed with coprolites were considered a result of chronic Chagas disease, whereas dilatation without coprolites was suggested as a result of post-mortem gas production [54]. In the present case, packing of the colon and rectum with feces was evident. Feces was bulging out the rectum without emerging at this site. Lack of relaxation of the internal sphincter of the anus in patients with chronic Chagas disease has been reported [49] and this seems to have lasted beyond the death of this child mummy.

### Lung infection

Adherence of both upper lobes of the lungs to the chest wall as well as the small lung and hilum calcifications on the right side indicated lung infection, possibly due to tuberculosis. In mummies, adherence of the lung to the chest wall is supposedly the result of a past episode of pneumonia with healing [13, 56]. Intact lungs are usually preserved in the form of a collapse to a different extent, depending on the mummification process. The calcifications could be the result of primary and/or postprimary pulmonary tuberculosis [57] however, they do not prove the diagnosis. Pulmonary calcifications can also occur in other infectious diseases, such as histoplasmosis or chicken pock pneumonia [57]. The adherence of the lungs could be the result of any infectious disease due to bacteria, viruses or fungi.

Until recently, it was thought that Europeans brought tuberculosis to America. Archaeological and paleopathological data succeeded in proving that the disease was already in America at least 2,000 years before the first contact from Europe [58–62]. A recent study on pre-Columbian mycobacterial genomes revealed seals as a source of New World human tuberculosis. The seals probably transmitted the disease to humans across the ocean [63]. This route would be in accordance with the fact that the coasts of Peru and northern Chile have long been recognized in the archaeological literature as locations where tuberculosis was first recognized in the New World [63]. It is reported that the Incas, with their small, dark, poorly ventilated one room houses, certainly sustained tuberculosis. Their well-organized system of communication probably helped to disseminate the disease amongst different people living within the powerful network of their empire [58]. Tuberculosis affects young people prior to and during the reproductive age [60, 62]. In conclusion, an infection of tuberculosis could be quite possible in the investigated child mummy.

Childhood mortality was reported to have been high in populations from coastal Peru and Chile. In most samples, nearly 50% percent of children died before 15 years of age [24]. Studies indicate that both chronic infectious disease, such as tuberculosis, and acute pulmonary infections were significant factors in morbidity and mortality in prehistoric Andean populations [24]. Additionally, the preserved surrounding subcutaneous fat of the entire mummy was rather sparse. Only minor skin folds were found, indicating that subcutaneous fat was absent at the time of death and not the result of post-mortem dehydration. Thus, the child could have been very thin by constitution or was cachectic as result of the suggested NF1, Chagas disease, and lung infection.

### Signs of violence

CT images revealed different findings that have to be discussed within the context of violence.

The cutaneous discontinuity on the right side of the neck was suggested to be the result of manipulation of the already mummified body, as the edges did not gape. However, a peri-mortem injury could not be definitely excluded.

The incision on the chest, however, must have occurred during the peri-mortem or early post-mortem phase, as this region would no longer have been accessible in the sitting position after rigor mortis had set in. A similar kind of incision was described by Verano [64] in five skeletons from a mass burial at Pacatnamu. In these individuals, the manubrium sterni were bisected by an oblique cut extending from the jugular notch infero-laterally to the region of the left first intercostal space. Additionally, multiple cervical fractures of the ribs were found. The observed pattern of mutilation led to the hypothesis that the incision was extended through the costal cartilages; this allowed swift and easy access to the organs of the thoracic cavity. However, the incision in the investigated child mummy was on the right side, and neither an involvement of the manubrium nor cervical fractures of the ribs were present. The population from the mass burial consisted of male adolescents and young adults, ranging in age from about 15 to 35 years with apparently good physical health. They were supposedly ritually sacrificed and mutilated before being deposited in the trench [64, 65]. Preservation of soft tissues in the present case revealed an additional large defect of the abdominal wall and displacement of the liver and parts of the feces outside of the abdomen. The thoracic and abdominal incisions seemed to be the result of an act of violence, apparently by a sharp weapon, with removal of some intra-abdominal contents. The liver seemed to have been cut into at least two major parts and was afterwards left outside the abdomen where it mummified, together with some of the displaced coprolites. Whether the absence of the lower parts of the heart was due to partial organ removal or decay remain unclear.

The additional fractures of the left humerus and tibia and the dislocation of some teeth could have occurred in the peri- or postmortem course.

In conclusion, the exact circumstances of the injuries, especially of the chest and abdomen, were not clear in this case. Hypotheses should include war, battle, murder, and accident [66]. Traumatic lesions in skeletons of Inca children were reported in the context of the heightened levels of violence following the Spanish invasion [67]. However, human sacrifice should also be considered [66], as discussed in the following paragraph.

### Wrapping, mummification, and cultural background

From the external view, the tunic was cut in two on the back, possibly indicating damage or manipulation. However, CT revealed an intact and very carefully performed inner wrapping of the mummy, comparable to the description of Inca bundles in the literature [68]. The shoe soles, strings and cords found between the textile layers were suggested to represent a pair of sandals. The small bags around the neck probably contained organic material, such as coca leaves or grains of maize.

The elaborate wrapping, including a spondylus shell and sandals, indicated a ceremonial burial of the child mummy [69]. Further information of the burial site or arrangement was not available in this isolated case.

The wrapping demonstrated the intention to preserve the child after bringing it into the desired position. However, any treatment of the body itself could not be found. The mummification seemed to be the result of dehydration due to the climate.

Child sacrifice has been documented in the Andes of Peru and is most clearly associated with the Inca Empire [66, 69–71]. For the so called “capacocha” interment at the top of mountains, children were chosen for reasons such as age, physical beauty (manifested by an immaculate skin) and social origin [66, 69]. However, it has been discoverd that Inca child sacrifice was not limited to mountaintops [68, 71]. Verano [66, 72] stated that there are two distinct patterns of human sacrifice identified at Prehispanic Peruvian sites: Firstly, carefully arranged burials of children or adolescents accompanied by elaborate grave goods; and secondly male captives buried in non-mortuary contexts without grave goods or considerate treatment of the body. Evidence of violence was found in the latter, but not in the former. It is important to mention that these reports are based mainly on skeletal findings. The child mummy in this study clearly revealed signs of violence, but these signs were found in the cartilage of the ribs and the abdominal wall and not in the skeleton. This means that well preserved non-bony and soft tissues revealed post-traumatic changes, which normally would have been lost in case of further decay. Another hypothesis to explain the death of this child, particularly the unusual, violent extraction of the liver while otherwise taking care to preserve the dead, could be organ divination. Divination, or the foreseeing of future outcomes, was practiced in the Inca culture by shamans. After a ritual killing of animals, such as llamas, the bodies of the animals were opened and organs were removed and cut into pieces to “read” their diagnosis [73, 74]. The mummified child was extremely unhealthy at the time of death. Based on CT findings the child was cachectic and probably stigmatized by cutaneous neurofibromas, suffered from digestion problems due to the assumed Chagas infection, and had suffered from lung infection. Perhaps this unusual child could have been seen as particularly suitable for such extreme divination processes.

## Conclusions

CT examination of the Inca bundle proved to be an important non-destructive examination method. CT images revealed a male child mummy with superior bony and soft tissue preservation. Standardized assessment, especially of the soft tissue structures, allowed for the diagnosis of NF1, the quite certain diagnosis of Chagas disease and the diagnosis of lung infection, probably due to tuberculosis. Additionally, the multi-morbid child seemed to have been cachectic at time of dead. The careful wrapping pointed to a ceremonial burial. Furthermore, CT revealed signs of fatal peri-mortem violence. Within the cultural background, hypothesis on the reason of this violence included war, battle, murder, accident, and human sacrifice.
